# Species conservation profiles of a random sample of world spiders I: Agelenidae to Filistatidae

**DOI:** 10.3897/BDJ.6.e23555

**Published:** 2018-04-25

**Authors:** Sini Seppälä, Sérgio Henriques, Michael L Draney, Stefan Foord, Alastair T Gibbons, Luz A Gomez, Sarah Kariko, Jagoba Malumbres-Olarte, Marc Milne, Cor J Vink, Pedro Cardoso

**Affiliations:** 1 Finnish Museum of Natural History, University of Helsinki, Helsinki, Finland; 2 IUCN SSC Spider & Scorpion Specialist Group, Helsinki, Finland; 3 Institute of Zoology, Zoological Society of London, London, United Kingdom; 4 Centre for Biodiversity and Environment Research, University College London, London, United Kingdom; 5 University of Wisconsin-Green Bay, Green Bay, United States of America; 6 University of Venda, Thohoyandou, South Africa; 7 University of Nottingham, Nottingham, United Kingdom; 8 Universidad Nacional de Colombia, Bogotá, Colombia; 9 Museum of Comparative Zoology, Harvard University, Cambridge, United States of America; 10 University of Barcelona, Barcelona, Spain; 11 University of Indianapolis, Indianapolis, United States of America; 12 Canterbury Museum, Christchurch, New Zealand

## Abstract

**Background:**

The IUCN Red List of Threatened Species is the most widely used information source on the extinction risk of species. One of the uses of the Red List is to evaluate and monitor the state of biodiversity and a possible approach for this purpose is the Red List Index (RLI). For many taxa, mainly hyperdiverse groups, it is not possible within available resources to assess all known species. In such cases, a random sample of species might be selected for assessment and the results derived from it extrapolated for the entire group - the Sampled Red List Index (SRLI). With the current contribution and the three following papers, we intend to create the first point in time of a future spider SRLI encompassing 200 species distributed across the world.

**New information:**

A sample of 200 species of spiders were randomly selected from the World Spider Catalogue, an updated global database containing all recognised species names for the group. The 200 selected species where divided taxonomically at the family level and the familes were ordered alphabetically. In this publication, we present the conservation profiles of 46 species belonging to the famillies alphabetically arranged between Agelenidae and Filistatidae, which encompassed Agelenidae, Amaurobiidae, Anyphaenidae, Araneidae, Archaeidae, Barychelidae, Clubionidae, Corinnidae, Ctenidae, Ctenizidae, Cyatholipidae, Dictynidae, Dysderidae, Eresidae and Filistatidae.

## Introduction

The IUCN Red List of Threatened Species is the most widely used information source on the extinction risk of species (Lamoreux et al. 2003, Rodrigues et al. 2006, Mace et al. 2008 but see Cardoso et al. 2011, Cardoso et al. 2012). It is based on a number of objective criteria, which are relatively easy to apply when adequate information is available (IUCN 2001). The Red List has been used to raise awareness about threatened species, guide conservation efforts and funding, set priorities for protection, measure site irreplaceability and vulnerability and influence environmental policies and legislation (Gardenfors et al. 2001, Rodrigues et al. 2006, Mace et al. 2008, Martín-López et al. 2009).

One of the uses of the Red List is to evaluate and monitor the state of biodiversity and a possible approach for this purpose is the Red List Index (RLI). The RLI helps to develop a better understanding of which taxa, regions or ecosystems are declining or improving their conservation status. It provides policy makers, stakeholders, conservation practitioners and the general public with sound knowledge of biodiversity status and change and tools with which to make informed decisions. The RLI uses weight scores based on the Red List status of each of the assessed species. These scores range from 0 (Least Concern) to 5 (Extinct/Extinct in the Wild). Summing these scores across all species, relating them to the worst-case scenario - all species extinct and comparing two or more points in time gives us an indication of how biodiversity is doing. At a global level, the RLI has been calculated for birds (Butchart et al. 2004, Hoffman et al. 2010), mammals (Hoffman et al. 2011), amphibians (Hoffman et al. 2010), corals (Butchart 2010) and cycads (United Nations 2015).

For many taxa, mainly hyperdiverse groups, it is not possible within available resources to assess all known species. In such cases, a random sample of species might be selected for assessment and the results derived from it extrapolated for the entire group - the Sampled Red List Index (SRLI, Baillie et al. 2008). The SRLI is now being developed for plants (Brummitt et al. 2015) and efforts towards a SRLI for butterflies (Lewis and Senior 2011) and Odonata are also in progress (Clausnitzer 2009).

Spiders currently comprise over 47000 species described at a global level (World Spider Catalog 2018). Of these, only 199 species (0.4%) have been assessed (www.redlist.org), of which the vast majority are from the Seychelles Islands or belong to the golden-orb weavers, Nephilidae (e.g. Kuntner et al. 2017). To these, a large number will be added in the near future, such as 55 species endemic to the Madeira and Selvagens archipelagos and 25 endemic to the Azores, all in Portugal (Cardoso et al. 2017, Borges et al. submitted). The vast majority of spiders assessed to date are therefore either regionally or taxonomically clustered and do not represent the group as a whole. With the current contribution and the three following papers, we intend to create the first point in time of a future spider SRLI encompassing 200 species distributed across the world.

## Methods

A sample of 200 species of spiders were randomly selected from the World Spider Catalog (2018), an updated global database containing all recognised species names for the group. The 200 selected species were divided taxonomically at the family level and those familes were ordered alphabetically. In this publication, we present the conservation profiles of 46 species belonging to the families alphabetically arranged between Agelenidae and Filistatidae, which encompassed Agelenidae, Amaurobiidae, Anyphaenidae, Araneidae, Archaeidae, Barychelidae, Clubionidae, Corinnidae, Ctenidae, Ctenizidae, Cyatholipidae, Dictynidae, Dysderidae, Eresidae and Filistatidae.

Species data were collected from all taxonomic bibliography available at the World Spider Catalog (2018), complemented by data in other publications found through Google Scholar and georeferrenced points made available through the Global Biodiversity Information Facility (www.gbif.org) and also other sources (https://www.biodiversitylibrary.org; https://login.webofknowledge.com; http://srs.britishspiders.org.uk; http://symbiota4.acis.ufl.edu/scan/portal; https://lepus.unine.ch; http://www.tuite.nl/iwg/Araneae/SpiBenelux/?species; https://atlas.arages.de; https://arachnology.cz/rad/araneae-1.html; http://www.ennor.org/iberia/). Whenever possible, with each species record, we also collected additional information, namely habitat type and spatial error of coordinates.

For all analyses, we used the R package red - IUCN redlisting tools (Cardoso 2017). This package performs a number of spatial analyses based on either observed occurrences or estimated ranges. Functions include calculating Extent of Occurrence (EOO), Area of Occupancy (AOO), mapping species ranges, species distribution modelling using climate and land cover information, calculating the Red List Index for groups of species, amongst others. In this work, the EOO and AOO were calculated in one of two ways:

- for range restricted species, for which we assumed knowledge of the full range, these values were classified as observed, the minimum convex polygon encompassing all observations used to calculate the EOO and the 2 km x 2 km cells known to be occupied and used to calculate the AOO. When the EOO was smaller than the AOO, it was made equal as per the IUCN guidelines (IUCN Standards and Petitions Subcommittee 2017).

- for widespread species or those for which we did not have confidence to know the full range, we performed species distribution modelling (SDM). This was done based on both climatic (Fick and Hijmans 2017) and landcover (Tuanmu and Jetz 2014) datasets, at an approximately 1x1 km resolution. Before modelling, the world layers were cropped to the region of interest to each species and reduced to four layers through a PCA to avoid overfitting. In addition, latitude and longitude were used as two extra layers to avoid the models predicting presences much beyond the known region following the precautionary principle. We then used the Maxent method (Phillips et al. 2006) implemented in the R package red. Isolated patches outside the original distribution polygon were excluded from maps to avoid overestimation of EOO and AOO values. All final maps and values were checked and validated by our own expert opinion. KMLs derived from these maps were also produced using the red package. The cells (2x2 km) predicted to be occupied were used to calculate the AOO. When the EOO was smaller than the AOO, it was made equal as per the IUCN guidelines (IUCN Standards and Petitions Subcommittee 2017).

To infer on possible changes in range and/or abundance and for forest species only, we have also consulted the Global Forest Watch portal (Global Forest Watch 2014), looking for changes in forest cover during the last 10 years that could have affected the species.

## Supplementary Material

Supplementary material 1 Distribution of *Coelotes amamiensis* Shimojana, 1989 Data type: Distribution
File: oo_170192.kml Cardoso, P.

Supplementary material 2 Distribution of *Hololena hopi* (Chamberlin & Ivie, 1942) Data type: Distribution
File: oo_170191.kml Cardoso, P.

Supplementary material 3 Distribution of *Oramia occidentalis* (Marples, 1959) Data type: Distribution
File: oo_170202.kml Cardoso, P.Cardoso, P.

Supplementary material 4 Distribution of *Amaurobius transversus* Leech, 1972 Data type: Distribution
File: oo_170203.kml Cardoso, P.

Supplementary material 5 Distribution of *Callobius pauculus* Leech, 1972 Data type: Distribution
File: oo_170204.kml Cardoso, P.

Supplementary material 6 Distribution of *Thaloe ennery* Brescovit, 1993 Data type: Distribution
File: oo_170205.kml Cardoso, P.

Supplementary material 7 Distribution of *Timbuka meridiana* (L. Koch, 1866) Data type: Distribution
File: oo_170206.kml Cardoso, P.

Supplementary material 8 Distribution of *Wulfila fragilis* Chickering, 1937 Data type: Distribution
File: oo_170207.kml Cardoso, P.

Supplementary material 9 Distribution of *Wulfila inornatus* (O. P.-Cambridge, 1898) Data type: Distribution
File: oo_170208.kml Cardoso, P.

Supplementary material 10 Distribution of *Alpaida alticeps* (Keyserling, 1879) Data type: Distribution
File: oo_170201.kml Cardoso, P.

Supplementary material 11 Distribution of *Alpaida veniliae* (Keyserling, 1865) Data type: Distribution
File: oo_170209.kml Cardoso, P.

Supplementary material 12 Distribution of *Araneus camilla* (Simon, 1889) Data type: Distribution
File: oo_170210.kml Cardoso, P.

Supplementary material 13 Distribution of *Araneus praedatus* (O. P.-Cambridge, 1885) Data type: Distribution
File: oo_174088.kml Cardoso, P.

Supplementary material 14 Distribution of *Cyclosa bianchoria* Yin et al, 1990 Data type: Distribution
File: oo_170212.kml Cardoso, P.

Supplementary material 15 Distribution of *Cyclosa nevada* Levi, 1999 Data type: Distribution
File: oo_170213.kml Cardoso, P.

Supplementary material 16 Distribution of *Cyrtarachne hubeiensis* Yin & Zhao, 1994 Data type: Distribution
File: oo_170214.kml Cardoso, P.

Supplementary material 17 Distribution of *Gea spinipes* L. Koch, 1843 Data type: Distribution
File: oo_170215.kml Cardoso, P.

Supplementary material 18 Distribution of *Larinia jeskovi* Marusik, 1987 Data type: Distribution
File: oo_170216.kml Cardoso, P.

Supplementary material 19 Distribution of *Mangora falconae* Schenkel, 1953 Data type: Distribution
File: oo_170217.kml Cardoso, P.

Supplementary material 20 Distribution of *Metazygia enabla* Levi, 1995 Data type: Distribution
File: oo_170218.kml Cardoso, P.

Supplementary material 21 Distribution of *Metazygia octama* Levi, 1995 Data type: Distribution
File: oo_170220.kml Cardoso, P.

Supplementary material 22 Distribution of *Metepeira ventura* Chamberlin and Ivie, 1942 Data type: Distribution
File: oo_170221.kml Pedro Cardoso

Supplementary material 23 Distribution of *Neoscona goliath* (Benoit, 1963) Data type: Distribution
File: oo_170222.kml Cardoso, P.

Supplementary material 24 Distribution of *Ocrepeida verecunda* Levi, 1995 Data type: Distribution
File: oo_170223.kml Cardoso, P.

Supplementary material 25 Distribution of *Plebs cyphoxis* (Simon, 1908) Data type: Distribution
File: oo_170224.kml Cardoso, P.

Supplementary material 26 Distribution of *Tatepeira itu* Levi, 1995 Data type: Distribution
File: oo_170225.kml Cardoso, P.

Supplementary material 27 Distribution of *Wagneriana yacuma* Levi, 1991 Data type: Distribution
File: oo_170226.kml Cardoso, P.

Supplementary material 28 Distribution of *Austrarchaea platnickorum* Rix & Harvey, 2011 Data type: Distribution
File: oo_170227.kml Cardoso, P.

Supplementary material 29 Distribution of *Zephyrarchaea marae* Rix & Harvey, 2012 Data type: Distribution
File: oo_170228.kml Cardoso, P.

Supplementary material 30 Distribution of *Aurecocrypta lugubris* Raven, 1994 Data type: Distribution
File: oo_170229.kml Cardoso, P.

Supplementary material 31 *Mandjela fleckeri* Raven & Churchill, 1994 Data type: Distribution
File: oo_170230.kml Cardoso, P.

Supplementary material 32 Distribution of *Nihoa kaindi* Raven, 1994 Data type: Distribution
File: oo_170231.kml Cardoso, P.

Supplementary material 33 Distribution of *Clubiona bifissurata* Kritscher, 1966 Data type: Distribution
File: oo_170232.kml Cardoso, P.

Supplementary material 34 Distribution of *Clubiona frisia* Wunderlich & Schuett, 1995 Data type: Distribution
File: oo_171382.kml Cardoso, P.

Supplementary material 35 Distribution of *Clubiona pyrifera* Schenkel, 1936 Data type: Distribution
File: oo_170234.kml Cardoso, P.

Supplementary material 36 Distribution of *Apochinomma nitidum* (Thorell, 1895) Data type: Distribution
File: oo_170235.kml Cardoso, P.

Supplementary material 37 Distribution of *Corinna venezuelica* (Caporiacco, 1955) Data type: Distribution
File: oo_170236.kml Cardoso, P.

Supplementary material 38 Distribution of *Acantheis variatus* Thorell, 1890 Data type: Distribution
File: oo_170237.kml Cardoso, P.

Supplementary material 39 Distribution of *Phoneutria keyserlingi* (F. O. P.-Cambridge, 1897) Data type: Distribution
File: oo_170238.kml Cardoso, P.

Supplementary material 40 Dsitribution of *Vulsor sextus* Strand, 1907 Data type: Distribution
File: oo_170240.kml Cardoso, P.

Supplementary material 41 Distribution of *Stasimopus nanus* Tucker, 1917 Data type: Distribution
File: oo_170241.kml Cardoso, P.

Supplementary material 42 Distribution of *Ilisoa hawequas* Griswold, 1987 Data type: Distribution
File: oo_170242.kml Cardoso, P.

Supplementary material 43 Distribution of *Argenna polita* (Banks, 1898) Data type: Distribution
File: oo_170243.kml Cardoso, P.

Supplementary material 44 Distribution of *Rhode baborensis* Beladjal & Bosmans, 1996 Data type: Distribution
File: oo_171412.kml Cardoso, P.

Supplementary material 45 Distribution of *Eresus kollari* Rossi, 1846 Data type: Distribution
File: oo_170748.kml Cardoso, P.

Supplementary material 46 Distribution of *Tricalamus jiangxiensis* Li, 1994 Data type: Distribution
File: oo_170245.kml Cardoso, P.
